# Spray-Dried Microcapsules of Cheese Whey and Whey Permeate as a Strategy to Protect Chia Oil from Oxidative Degradation

**DOI:** 10.17113/ftb.60.04.22.7451

**Published:** 2022-12

**Authors:** Marcos Aurélio Dahlem Júnior, Wendell Dall Agnol, Natália Neitzke, Adriani Cristina Felipe dos-Santos, Vanessa Mendonça Esquerdo, Daniel Neutzling Lehn, Luiz Antonio de Almeida Pinto, Claucia Fernanda Volken de Souza

**Affiliations:** 1 Laboratory of Food Biotechnology, Graduate Program in Biotechnology, University of Vale do Taquari (Univates), Avelino Tallini Avenue, 171, Postal code 95914-014, Lajeado, RS, Brazil; 2 Industrial Technology Laboratory, School of Chemistry and Food, Federal University of Rio Grande, FURG, Italia Avenue, km 08, Postal code 96203-900, Rio Grande, RS, Brazil

**Keywords:** whey permeate, cheese whey, chia oil, microcapsules

## Abstract

**Research background:**

Cheese whey and whey permeate are dairy industry by-products usually sent to effluent treatment or incorrectly disposed in the environment, generating costs for the production of dairy products and environmental problems due to the high organic load. Cheese whey and whey permeate can be reused as wall materials to form chia oil microcapsules, which act as a barrier to prooxidants. This study aims to develop an encapsulation method by spray-drying to protect chia oil using dairy by-products as wall materials.

**Experimental approach:**

We evaluated cheese whey, whey permeate and mixtures of *m*(cheese whey):*m*(whey permeate)=50, 70 and 80% as encapsulating agents with the spray-drying process. Initially, we characterized the chia oil and encapsulating materials. Chia oil emulsions were prepared using the encapsulating materials and an emulsifier. The stability of the emulsions was evaluated by creaming index, and they were characterized according to size distribution and polydispersity index. Emulsions were encapsulated in a spray dryer with inlet and outlet air temperature at 125 and 105 °C, respectively. After encapsulation, we assessed the oxidative degradation of chia oil over 30 days of storage by determining the peroxide index.

**Results and conclusions:**

Emulsions presented creaming index between 51 and 83% in all formulations, and the oxidative stability of microencapsulated chia oil was significantly higher than that of free chia oil after 30 days. Wall material combination affected both encapsulation efficiency and oxidation protection. The cheese whey and whey permeate (8:2) mixture exhibited the highest encapsulation efficiency (70.07%) and ability to protect the chia seed oil. After 30 days, the peroxide value was below the maximum limit considered safe for human consumption.

**Novelty and scientific contribution:**

According to these results, dairy by-products can be used for encapsulation of oxidation-sensitive oils. This represents an alternative use for dairy by-products, which otherwise are discarded and can impact the environment due to their high organic load. Our findings suggest that dairy by-products can be effectively used as wall materials to generate value-added products.

## INTRODUCTION

Chia (*Salvia hispanica* L.) is a plant species from the *Lamiaceae* family, native to Latin America. Approximately 28 to 32% of its oil is found in the seed. The oil has high levels of polyunsaturated fatty acids (PUFA), especially ω-3 and ω-6, with ω-3 representing about 61 to 70% of total oil content. This plant is one of the richest plant sources of ω-3 and provides a highly nutritive seed oil (*1*, *2*). Therefore, chia oil can be employed to develop functional foods for a diet rich in ω-3, being an alternative to fish for vegetarians (*3*). However, chia seed oil has low stability and it is susceptible to oxidation, thus reducing its shelf life (*1*, *2*). The exposure of PUFA to factors such as oxygen, moisture and temperature triggers reactions that cause oil to deteriorate, thus limiting its application in foods (*4*, *5*).

Encapsulation technologies can minimize chia oil degradation. Oil-in-water emulsions form the basis of numerous products. They enhance the physical properties of the oil and facilitate encapsulation and preservation (*6*). Microencapsulation is the packaging of small particles (active substance) by wrapping them with a homogeneous matrix, forming small capsules (*7*). One of the most commonly applied encapsulation technologies is spray drying, which is fast, relatively inexpensive and reproducible. Moreover, constant drying conditions stabilise powder specifications throughout the dryer. As it is a continuous operation and adaptable to fully automatic control, it can be used to dehydrate heat-sensitive materials (*8*-*11*).

The choice of wall material is of utmost importance, as it determines microcapsule efficiency and stability (*12*, *13*). Cheese whey and whey permeate, natural and edible biopolymers, may be alternatives for oil encapsulation. A large volume of cheese whey is generated in the cheese making process, ranging from 80 to 90% of the used milk volume (*13*). Between 2017 and 2019, more than 230 000 kt of cheese whey were produced. Global whey production is estimated to reach approximately 268 000 kt annually by 2029 (*14*). That means large amounts of cheese whey are available from dairy industry processes and can be reused instead of simply being discarded, as it has a high organic loading rate. Cheese whey is an environmental pollutant that cannot be discarded without previous treatment (*14*, *15*). Alternatively, it can be ultrafiltered to obtain whey retentate, a product rich in proteins with high added value. However, this process generates another liquid waste, whey permeate, comprised mostly of lactose, with high organic loading (*15*-*18*).

The literature shows studies on lipid oxidation of the oil of different origins, such as anchovy (*19*), pomegranate seed (*20*), carp (*21*) and fish oil (*22*, *23*). Studies on chia oil protection are still recent, and there is no study on cheese whey and its permeate efficiency in microencapsulation of chia seed oil. Therefore, further studies of the production of stable and efficient chia oil microcapsules prepared with these dairy by-products are required. Using cheese whey and whey permeate as wall materials adds value to these by-products and can reduce environmental effects of incorrect disposal. Thus, this study aims to evaluate the use of dairy by-products as wall materials for chia oil powders using spray drying. We evaluated the influence of different cheese whey and whey permeate ratios in wall materials on chia seed oil emulsion stability, encapsulation efficiency and oxidative stability.

## MATERIALS AND METHODS

### Materials and characterisation

Cheese whey and whey permeate (powdered) were donated by regional dairy factories and characterized following the methods of the Association of Official Agricultural Chemists (AOAC) for lipid (2000.18), protein (991.20), moisture (990.20), carbohydrate (986.25) and ash contents (968.08) (*24*).

The chia seed oil (Girioil Agroindústria, Entre-Ijuís, Brazil) was characterized according to the methods of the American Oil Chemists Society (AOCS) for iodine (Cd 1–25), acid (Cd 3d–63) and saponification values (Cd 3b–76) (*25*). Peroxide value was determined according to a method adapted from literature (*26*). Briefly, an aliquot of 0.05 g of oil was weighed, and a volume of 9.85 mL of a chloroform/methanol 7:3 solution was added, along with 50 mL iron(II) chloride and 50 mL ammonium thiocyanate. The sample was shaken for 10 s after each solution was added. The reaction was conducted in the absence of light for 5 min, and the absorbance was measured at 500 nm in a spectrophotometer (UV-2600; Shimadzu, Tokyo, Japan). Fatty acid composition was determined by gas chromatography (GCMS-QP2010; Shimadzu) according to literature (*27*). We used gum arabic (Labsynth, Diadema, Brazil) and soy lecithin (Bremil, Arroio do Meio, Brazil) as emulsification agents. All used reagents were of analytical grade.

### Preparation and characterisation of chia oil-in-water emulsion

Five emulsions were prepared using different wall materials in aqueous solutions. First, the gum arabic was dissolved (3.0% *m*/*V*) with distilled water at 60 °C. The wall material was added (23.0% *m*/*V*) at 25 °C. Cheese whey, whey permeate and mixtures of *m*(cheese whey):*m*(whey permeate)=50, 70 and 80% *m*/*m*) were used as wall materials. The solution was constantly agitated at 300 rpm for 6 h at 25 °C in an orbital shaker incubator (MA 830; Marconi, Piracicaba, Brazil), according to the method adapted from literature (*28*). The oil phase, chia oil (9.28% *m*/*V*) and soy lecithin (0.5% *m*/*V*) were added to the initial solution. Emulsions were obtained by phase homogenization using an Ultra–Turrax homogenizer (Ultra-Turrax S25N18GST; IKA, Campinas, Brazil) for 5 min at 8000 rpm. The procedure and conditions were selected according to Lehn *et al*. (*29*).

Emulsion stability was determined using creaming index (CI), as proposed in the literature (*30*). Emulsions were transferred to 10-mL test tubes and kept in a drying oven at 25 °C for 24 h. CI was determined using the emulsion height, according to the following equation:



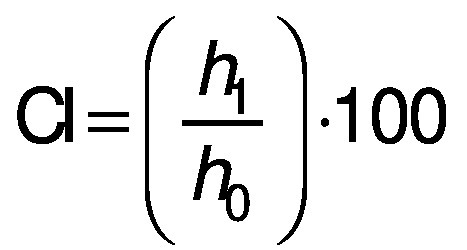



where *h*_0_ is the initial height of the emulsion (cm), and *h*_1_ is the height of the whey-rich (upper) phase after 24 h (cm).

Emulsions were characterized according to size distribution and polydispersity index (PDI) by dynamic light scattering (Litesizer 500; Anton Paar, São Paulo, Brazil). Morphology was observed using an optical microscope (DM 500; Leica, Wetzlar, Germany) with 40× magnification.

### Spray-drying process

Emulsions were spray-dried immediately after preparation using a lab scale spray-dryer (MD 0.5; LabMaq, Ribeirão Preto, Brazil) with inlet and outlet drying temperatures of 125 and 105 °C respectively, feed rate of 300 mL/h, two-fluid nozzle (0.7 mm diameter), and drying air and spray flow rates of 2.5 m^3^/min and 45 L/h, respectively (*12*).

### Microcapsule characterisation

The surface oil content, total oil content and encapsulation efficiency of the microcapsules were characterized according to the method proposed in the literature (*12*). The amount of unencapsulated oil was measured by adding hexane (15 mL) to 2 g of microcapsule powder and shaking for 2 min. The suspension was filtered, and the residue was rinsed three times with 20 mL of hexane each time. The filtrate solution containing the extracted oil was then transferred to an oven at 70 °C where it remained for 6 h. Surface oil was calculated by the difference between the initial and final slurry container mass, and encapsulation efficiency (EE) was calculated using the following equation:



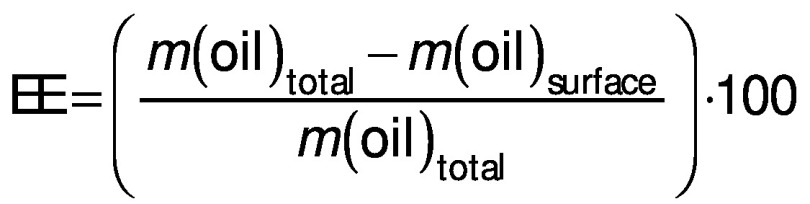



Water activity (*a*_w_) was measured using the AquaLab system (model Lite; Meter Group Latam, São José dos Campos, Brazil) at 25 °C. Hygroscopicity values of the microcapsules were determined according to a method described in the literature (*31*). Briefly, the powder samples (approx. 1 g) of each treatment were placed in a container with saturated NaCl solution at 25 °C. After one week, the samples were weighed and hygroscopicity was expressed in g of adsorbed moisture per 100 g dry solids. Colour parameters were determined using a colorimeter (CM–5; Minolta Corporation, Tokyo, Japan) by measuring the three-dimensional Lab colour space (*32*).

The presence of functional groups of chia seed oil in the microcapsules was determined using Fourier transform infrared spectroscopy (FTIR; 4100 ExoScan; Agilent Technologies, Santa Clara, CA, USA). Scanning electron microscopy (SEM) was used for morphological analysis and average size determination of microcapsules. The samples were placed on stainless steel stubs and sputter-coated with gold at 20 kV, and then observed under the microscope (EVO MA15; Carl Zeiss, Oberkochen, Germany) at 300 to 5000× magnification.

The stability of microencapsulated chia seed oil with cheese whey, its permeate, and mixtures of *m*(cheese whey):*m*(whey permeate)=50, 70 and 80% was monitored at 25 °C, protected from light, for 30 days. Peroxide values of microcapsules and free chia oil were evaluated to analyse the protective effect of the encapsulating agents. A method was adapted to extract oil microcapsules (*33*). A microcapsule powder sample (1 g) was suspended in 1 mL of distilled water and stirred for 30 min in an incubator (MA830; Marconi, Piracicaba, Brazil) at 300 rpm and 20 °C. A 0.6-mL aliquot of this solution was homogenized with 1.5 mL of isooctane/isopropanol (2:1) solution. The supernatant was collected after centrifugation at 1000×*g* (Universal 320R; Andreas Hettich GmbH & Co. KG, Tuttlingen, Germany) and rinsed with the solvent three times. An aliquot (0.5 mL) of the extracted product was used for peroxide value analysis. Peroxide content in free and encapsulated chia seed oil after 0, 3, 7, 15 and 30 days was analysed according the literature (*26*). An aliquot of 0.05 g of oil was weighed, and a volume of 9.85 mL of a chloroform/methanol 7:3 solution was added, along with 50 mL iron(II) chloride and 50 mL ammonium thiocyanate. The sample was shaken for 10 s after each solution was added. The reaction was conducted in the absence of light for 5 min and the absorbance was measured at 500 nm in a spectrophotometer (UV-2600; Shimadzu). The measurements were performed in triplicate. The hydroperoxide concentration was determined using a standard curve of Fe(III) ranging from 1 to 30 mg. The peroxide value was expressed in mmol per kg oil.

### Statistical analyses

Encapsulation experiments and analytical determinations were conducted in triplicate. Statistical data were analysed using the analysis of variance (ANOVA). Mean values were compared using the Tukey’s test at a significance level of 95% (p≤0.05) using Statistica® 7.1 software (*34*).

## RESULTS AND DISCUSSION

### Characterisation of dairy by-products

Based on the cheese whey and whey permeate (powdered) chemical compositions, moisture in the wall materials was approx. 3.0%, ash content 5.0%, and lipid content <0.5%. However, both materials had different compositions depending on the production process. One particular difference between the cheese whey and its permeate is protein amount. Whey permeate had lower protein content (1.5%) than cheese whey (10.9%) since proteins are retained in the membrane during ultrafiltration of cheese whey, which consequently increases carbohydrate content. Carbohydrate content, primarily consisting of lactose, was 90.1% in whey permeate and 78.5% in cheese whey (*35*, *36*). Cheese whey proteins enable emulsification, important in oil microencapsulation (*37*). This suggests that cheese whey performs better as wall material.

### Characterisation of chia seed oil

Table 1 shows the physicochemical characterisation of chia seed oil. The major fatty acids present in chia seed oil determined by gas chromatography (GC) analysis were palmitic, C16:0, and stearic, C18:0 (10.9%), oleic, C18:1 (6.7%), linoleic, C18:2 (17.1%) and linolenic, C18:3 (64.2%) acids. The high PUFA content (82.4% total) indicates that chia seed oil is highly unsaturated, corroborated by the high iodine value (Table 1). This unsaturation degree makes chia seed susceptible to oxidation, particularly when exposed to adverse factors such as light, oxygen, moisture and temperature (*4*, *5*).

**Table 1:** Physicochemical characterisation of commercial chia seed oil

Characterisation index	Chia seed oil
Iodine value *w*(I_2_)/(g/100 g)	173.4±5.3
Saponification value *w*(KOH)/(mg/g)	196.5±2.8
Peroxide value (*n*(O_2_)/*m*(oil))/(mmol/kg)	4.0±0.2
Acid value *w*(oleic acid)/(mg/100 g)	0.34±0.04

Mean value±standard deviation (*N*=3)

Chia seed oil had a high saponification value (*38*) (Table 1), which is inversely proportional to fatty acid chain length, *i.e.* the index tends to be higher in oils with a higher number of short-chain triglycerides (*39*). Free fatty acids and peroxide values are parameters used to determine the quality of the oil. There is specific legislation to determine the maximum limits of these indices to ensure food quality. Free fatty acids and peroxides in oil (Table 1) were within the limit established by CODEX Alimentarius (*40*), which should be lower than *w*(oleic acid)=0.6 mg/100 g and (*n*(O_2_)/*m*(oil))=10 mmol/kg, respectively.

### Characterisation of emulsions

The CI (after 24 h of storage), size distribution, and PDI of the emulsions are shown in Table 2. None of the emulsions was particularly resistant to cream formation, and during storage, the emulsions separate into an opaque white layer at the top and a cloudy layer at the bottom. According to Owens *et al*. (*41*), a low repulsive electrostatic force between droplets leads to rapid creaming. It is common for polysaccharides and proteins to form interactions at the pH value between the isoelectric point (pI) of the protein and the p*K*a of the polysaccharide.

**Table 2:** Effect of wall materials on the characteristics of chia oil-in-water emulsions

Sample	Creaming index/%	*d*/µm	Polydispersity index
Cheese whey (CW)	(51.7±1.2)^c^	(3.2±0.2)^b^	(0.27±0.04)^a,b^
Whey permeate (WP)	(83.5±0.4)^a^	(4.0±0.2)^a^	(0.25±0.01)^b^
(*m*(CW):*m*(WP))/%			
50	(82.1±0.3)^b^	(3.7±0.2)^a^	(0.31±0.03)^a^
70	(80.3±1.1)^c^	(4.0±0.2)^a^	(0.29±0.03)^a^
80	(78.1±1.2)^d^	(3.9±0.3)^a^	(0.29±0.02)^a^

Mean value±standard deviation (*N*=3). Different letters in the same column indicate a significant difference (p<0.05)

In addition, Table 2 shows that the highest CI values are obtained for the capsules that used pure whey permeate or its highest mass fractions (50 and 70%). These differences in CI may be correlated with protein content in the emulsion. Proteins facilitate the formation of a stable interface between the oil and the aqueous phase. Milk proteins adsorb the oil-water interface, forming micelles that coat the oil phase (*41*, *42*). Noello *et al*. (*43*) studied the emulsion of chia seed oil using whey protein concentrate and pectin. They observed that the emulsion containing only whey protein concentrate remained stable for 24 h, indicating the ability of whey proteins to stabilise the emulsion.

**Fig. 1:**
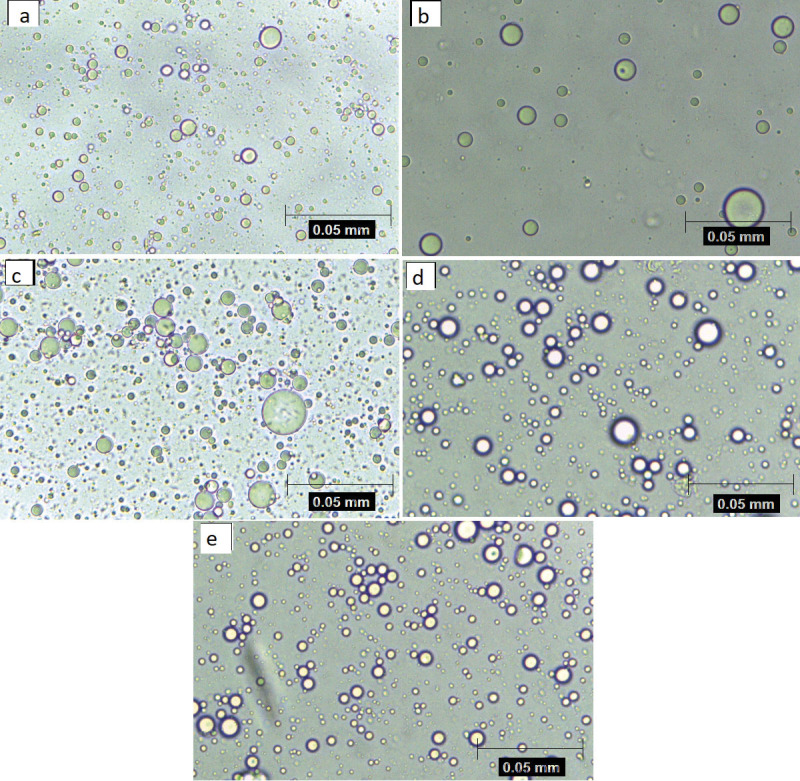
Images of chia oil emulsions at 40× magnification. Wall materials: a) cheese whey (CW), b) whey permeate (WP), c-e) *m*(CW):*m*(WP)=50, 70 and 80% respectively

CI can provide indirect information on the extent of droplet aggregation in an emulsion: the higher the CI, the larger the droplets and/or the higher the aggregation (*44*), confirmed by size distribution and high PDI values (above 0.2), which indicate a tendency towards agglomeration (Table 2). These values are confirmed in Fig. 1, which shows the morphology of the emulsions. Fig. 1 correlates diameter, uniformity and distribution of emulsion droplets to emulsion stability, and consequently, to the quality of the microcapsules (*45*). Emulsions were spray-dried to check the viability of dairy wall materials to prepare microcapsules and protect the chia oil, considering that wall materials can create a physical barrier to the air, without chemical interactions with the core oil.

### Characterisation of microcapsules

Table 3 shows encapsulation efficiency (EE/%), water activity (*a*_w_) and hygroscopicity (%) of samples. Based on the results, it is evident that the EE is directly related to the type of wall material used. The highest value (~70%) was obtained using the mixture of 80% cheese whey and 20% whey permeate, indicating good encapsulation. Adding fractions of permeate to the wall material decreases the hydrophobicity of whey proteins, preventing the migration of hydrophobic compounds from the core material to the microcapsule surface (*46*). Rodea-González *et al*. (*47*) encapsulated chia seed oil using spray-drying with cheese whey concentrate and gum arabic, and obtained an encapsulation efficiency of 70%. Gallardo *et al*. (*5*) microencapsulated flaxseed oil using cheese whey isolate, maltodextrin and gum arabic as wall materials and obtained ~87% of encapsulation efficiency. The EE results shown in Table 3 indicate that cheese whey and whey permeate perform as well as the whey concentrates and isolates used in the mentioned studies.

**Table 3:** Characterisation parameters of chia seed oil microcapsules

Wall material	Encapsulation efficiency/%	Water activity	Hygroscopicity/%
Cheese whey (CW)	(56.7±2.6)^b^	(0.11±0.04)^b^	(9.2±0.2)^a^
Whey permeate (WP)	(29.5±0.6)^d^	(0.24±0.06)^a^	(3.70.4)^d^
(*m*(CW):*m*(WP))/%			
50	(44.4±2.9)^c^	(0.22±0.03)^ab^	(5.5±0.8)^c^
70	(43.2±4.3)^c^	(0.26±0.06)^a^	(5.9±0.6)^c^
80	(70.1±1.1)^a^	(0.20±0.03)^ab^	(7.2±0.1)^b^

Different letters in the same column indicate significant difference (p≤0.05)

Water activity (*a*_w_) indicates the amount of water available for microbiological development and degradation reactions. Table 3 shows that the water activity of microcapsule powders was lower than 0.3, within the range for atomized products (*48*). The ability to absorb water from the environment indicates hygroscopicity, which is directly associated with the preservation of the material. Products with low hygroscopicity can be preserved for longer time (*31*). Hygroscopicity values were higher in the experiments with higher whey content (CW and mixture with 80% CW; Table 3). This difference is associated with wall material composition. During emulsification and drying, lactose derived from both cheese whey and whey permeate can assume different forms since there is no lactose crystallization before drying. According to Hargrove *et al*. (*49*), lactose crystallization, which may occur during drying, reduces the hygroscopicity in whey powder, thus explaining the reduced hygroscopicity in wall materials that have higher lactose content. The lactose content of cheese whey was approx. 50%, and that of whey permeate 76%. The stability of these powders depends on hygroscopicity, which is influenced by the amount of lactose form: alpha form (less hygroscopic) or beta (more hygroscopic).

**Fig. 2:**
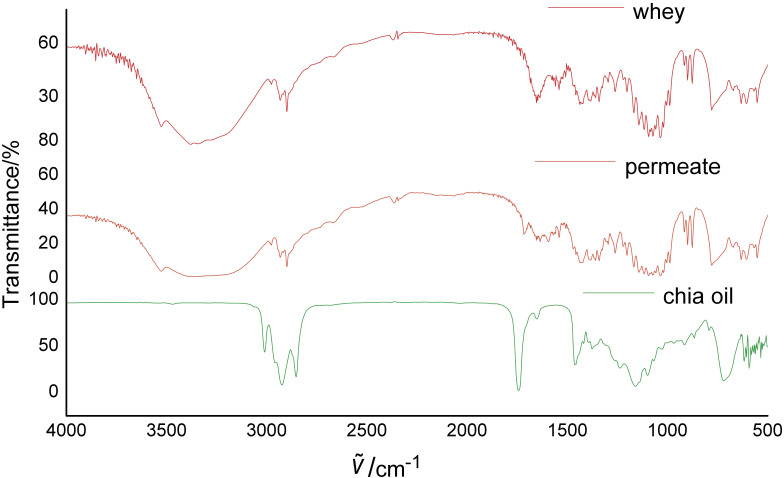
Infrared spectrum of chia seed oil, whey permeate and cheese whey

Fig. 2 shows the FTIR spectrum of chia seed oil and dairy by-products. Some bands at 3430, 1383, 1296, 1260, 1142, 1117, 1035, 915, 898 and 632 cm^–1^ are typical of lactose, with its high content in cheese whey (75%) and whey permeate (85%) (*50*). There was a peak at 3100 cm^–1^ attributed to a deformation associated with a hydrogen bound to an unsaturated fatty acid carbon. The bands at 2920 and 2850 cm^–1^ represented the –CH_2_– symmetric and asymmetric stretching vibrations, respectively. The double bond between carbon and oxygen (C=O) was represented at 1740 cm^–1^. There was a tendency towards forming a band near 1250 cm^–1^ associated with the C–O bond in the esters of fatty acids. The band near 950 cm^–1^ was associated with the overlapping of out-of-plane angular vibrations of the –HC=CH– (*cis* and *trans*) groups (*51*). According to the spectrum of microcapsules (not shown), the nature of the peaks did not vary in the oil combined with wall material, which indicates lack of any significant chemical interactions between them. All the microcapsule spectra were similar, as the main bonds of the used materials were similar, and no new bonds were formed *via* chemical reactions. This indicates the absence of chemical interactions between the components of the microcapsules.

Table 4 shows data on the colour of microcapsules with different wall materials. Cheese whey in its original form had a hue angle of 85.3° and chromaticity of 13.9, indicating a tendency towards light yellow coloration. Whey protein had a hue angle of 72.3° and a chroma of 26.1, and therefore a darker brown coloration. Chroma values indicate colour intensity, which increased with higher content of whey protein in the wall material, probably due to reactions such as caramelization and Maillard reaction related to emulsion drying. Even so, the capsules did not show intense coloration. Furthermore, hue values between 70 and 90° refer to yellow coloration, which was expected considering the applied wall material. The experiment conducted with whey permeate and the mixture of 50% cheese whey and 50% its permeate showed a significant change in hue angle, associated with the wall material. Therefore, the relationships between *H°* and C* lead to the conclusion that the produced capsules are yellow-whitish, although darker hues may be obtained by darkening reactions due to heating during drying.

**Table 4:** Colour parameters of microcapsules

Wall material	L*	a*	b*	C*	*H*/*°*
Cheese whey (CW)	(85.23±0.01)^a^	(3.84±0.02)^c^	(23.35±0.02)^c^	(23.33±0.03)^a^	(80.72±0.01)^c^
Whey protein (WP)	(68.27±0.02)^c^	(7.73±0.01)^a^	(29.12±0.01)^a^	(30.28±0.02)^c^	(75.13±0.03)^a^
(*m*(CW):*m*(WP))/%					
50	(74.22±0.02)^b^	(5.87±0.02)^b^	(26.96±0.01)^ab^	(26.80±0.01)^b^	(75.33±0.02)^a^
70	(73.24±0.01)^b^	(4.9±0.0)^bc^	(25.31±0.01)^bc^	(26.00±0.00)^ab^	(79.27±0.00)^bc^
80	(76.68±0.02)^b^	(4.68±0.02)^c^	(25.16±0.03)^bc^	(25.44±0.04)^a^	(79.63±0.03)^bc^

Mean value of three independent experiments±standard deviation. Different letters in the same column indicate significant difference (p≤0.05)

**Fig. 3:**
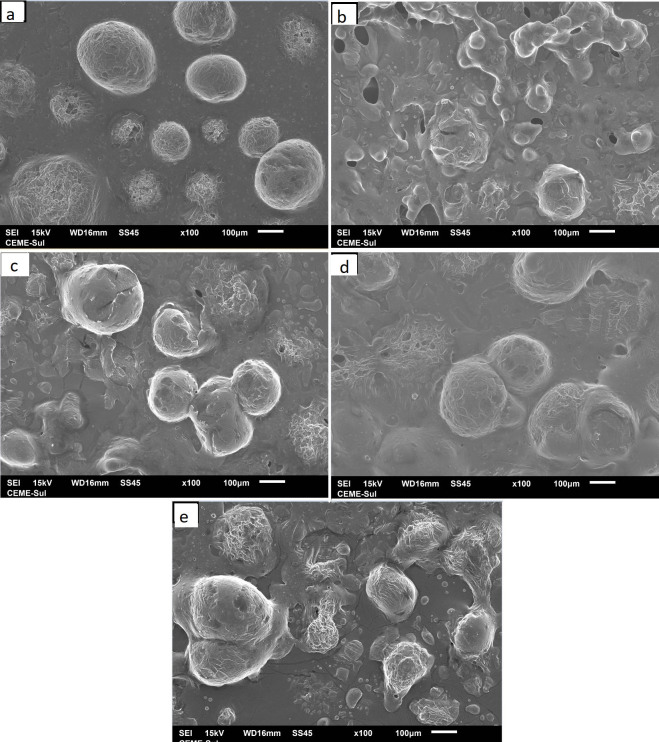
Scanning electron microscope images of microcapsules with: a) cheese whey (CW), b) whey permeate (WP), c-e) *m*(CW):*m*(WP)=50, 70 and 80% respectively

Fig. 3 shows the SEM images of the microcapsules obtained with dairy by-products and their mixtures. Fig. 3a indicates that chia oil microencapsulated with cheese whey has a greater tendency to have a spherical shape than whey permeate and the cheese whey and whey permeate mixtures. Fig. 3 does not indicate fissures and broken microcapsules. The difference between amounts of microcapsule components, such as lactose and protein, may be associated with the behaviour shown in the formation and distribution of these microcapsules. The aggregation observed in Fig. 3b could be explained because whey permeate has a higher lactose content than cheese whey, contributing to adhesiveness effects due to exposure to the environment. Lactose, depending on its content and form in the product, may provide higher hygroscopicity to the powder. Humidity and low-molecular-mass sugars, such as lactose, are the main sources of instability of spray-dried milk powders.

**Fig. 4:**
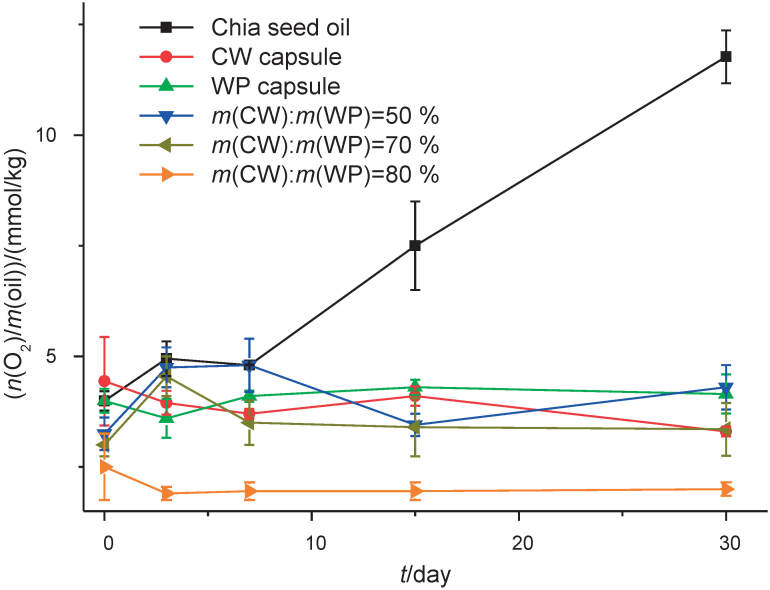
Stability of free commercial and microencapsulated chia seed oil for 30 days at 25 °C. CW=cheese whey, WP=whey permeate

The oxidative stability of microencapsulated chia oil can be observed in Fig. 4 (storage at 25 °C). Fig. 4 shows positive effects of encapsulation on the stability of chia oil during storage. Free chia oil had increased peroxide value. These results indicate a relationship between encapsulation efficiency and the maintenance of peroxide values in the microcapsules. The mixture of 80% cheese whey and 20% whey permeate had higher encapsulation efficiency, providing higher oxidation resistance and stability. Furthermore, the wall material provided better protection for the encapsulated oil after drying, which was the primary reason for changes in peroxide value compared to free oil on the initial day. Due to their flexibility and amphiphilic nature, milk proteins rapidly adsorb the emulsion interface, where they self-aggregate and form continuous and homogeneous membranes around oil droplets through intermolecular beta sheet interactions. By coating oil droplets with charged layers, protein films provide an electrostatic barrier against flocculation and coalescence, allowing the formation of efficient microcapsules after spray drying (*52*). Possibly the surface oil is responsible for the first result (in the case of microcapsules with the mixture of 80% cheese whey and 20% whey permeate) of the peroxide value, as an effect of the exposure to the drying temperature. The other particles collected from day 3 onwards were less exposed to air.

The use of cheese whey and whey permeate in emerging technologies such as microencapsulation represents an alternative and beneficial use of these low-cost waste products with high organic load. Moreover, microcapsules formed with these dairy by-products as wall materials can be used to develop high value-added food.

## CONCLUSIONS

Combinations of dairy by-products (cheese whey and whey permeate) were used to microencapsulate chia oil by spray-drying. Cheese whey with low mass fractions of whey permeate was a good alternative as additional wall material to form emulsions. Higher values of creaming index found in the capsules with the highest amounts of whey permeate were related to larger particle diameter. The powder was spherical, with low water activity and hygroscopicity, which is typical of microcapsules formed using spray-drying. The oxidative stability of microencapsulated chia oil was significantly higher than that of free chia oil after 30 days in all formulations. Wall material combination affected both encapsulation efficiency and oxidation protection. The mixture of 80% cheese whey and 20% whey permeate had the highest encapsulation efficiency and ability to protect the chia seed oil. The process generated microcapsules with positive effects in protection against lipid oxidation. These results indicate the viability of using dairy by-products as wall materials to generate value-added products.
